# Back-translation effects on static and contextual word embeddings for topic classification embedding in classification tasks

**DOI:** 10.1371/journal.pone.0330622

**Published:** 2025-08-29

**Authors:** Dávid Držík, Lívia Kelebercová

**Affiliations:** Department of Informatics, Faculty of Natural Science and Informatics, Constantine the Philosopher University in Nitra, Nitra, Slovakia; American University of Beirut, LEBANON

## Abstract

This study investigates the impact of back-translation on topic classification, comparing its effects on static word vector representations (FastText) and contextual word embeddings (RoBERTa). Our objective was to determine whether back-translation improves classification performance across both types of embeddings. In experiments involving Logistic Regression, Support Vector Machine (SVM), Random Forest, and RNN-LSTM classifiers, we evaluated original datasets against those augmented with back-translated data in six languages. The results demonstrated that back-translation consistently enhanced the performance of classifiers using static word embeddings, with the F1-score increasing by up to 1.36% for Logistic Regression and 1.58% for SVM. Random Forest saw improvements of up to 2.80%, and RNN-LSTM by up to 1.46%; however, these gains were smaller in most languages and did not reach statistical significance. In contrast, the effect of back-translation on contextual embeddings from the RoBERTa model was negligible: no language showed a statistically significant F1-score improvement. Despite this, RoBERTa still delivered the highest absolute performance, suggesting that advanced contextual models are less reliant on external data augmentation techniques. These findings indicate that back-translation is especially beneficial for classification tasks in low-resource languages when using static word embeddings, but its utility is limited for modern context-aware models.

## Introduction

Text Data augmentation includes techniques used to increase the amount of data by adding modified copies of already existing data or newly created synthetic data [1]. In natural language processing (NLP) tasks, various text augmentation techniques are used for small datasets or unbalanced datasets to improve model performance. Augmentation of datasets is useful in the case of low-resource languages, where the availability of text datasets is often limited. Data augmentation techniques can be divided according to several taxonomies [2]. One of the common taxonomy is to divide them into the synonym replacement [3], mix-up based techniques [4], and generative techniques [5]. Another well-known taxonomy applicable in NLP tasks is mentioned by authors [2,6,7] who divided the techniques into the character level, word level, phrase and sentence level, and document level.

One of the augmentation techniques is back-translation which is usually done on phrase and sentence level, or document level. Back-translation is a method that can create more training data without needing extra labels. It works by translating text into a different language and then back again, creating different versions of the same original text. This helps to increase the amount of data available for training. This technique introduces small variations in text that can make models more robust by exposing them to different paraphrased versions of the same content. In this way, it is possible not only to retain the meaning but also to ensure syntactic diversity [2], unlike methods such as substructure substitution [8] and syntactic shortening [9]. While the use of back-translation has been studied in tasks such as machine translation [10] and text generation [11], its impact on word embedding both static and contextual remains understudied.

Word embeddings provide dense vector representation of words capturing semantic and syntactic relationships. Recent advancements in word embeddings have revolutionized this domain, with two prominent types of embeddings playing key roles: static word embeddings, such as Word2Vec [12], FastText [13], and GloVe [14], and contextual word embeddings, such as BERT [15] and GPT-based [16] models. Static embeddings, such as Word2Vec or FastText assign a single fixed vector to each word regardless of context. On the other hand, contextual embeddings, such as BERT, GPT generate contextual representations based on the surrounding text.

This study focuses on understanding whether the impact of back-translation on classification performance will differ between static and contextual word embeddings in a low-resource language. While various static and contextual embeddings exist, in this work we focus on FastText [13] as the static word embedding and RoBERTa [17] as the contextual word embedding to provide a clear and focused comparison relevant for Slovak as a low-resource language [18]. Static embeddings, by their nature, represent words with fixed vectors, meaning that small perturbations in word choice or phrasing caused by back-translation could lead to shifts in the representation of words. To clarify, we are focusing on back-translating the classification dataset itself. The goal is to evaluate whether back-translation improves classification performance when using both static and contextual word vectors. Specifically, we hypothesize that static word vectors, which inherently lack the ability to adjust dynamically to context, will exhibit more significant differences between original and back-translated data compared to contextual embeddings. This distinction arises directly from the fundamental characteristics of these embedding types. Static embeddings, such as FastText, assign a single, fixed vector representation to each word, regardless of its surrounding words. Consequently, even subtle lexical or syntactic variations introduced through back-translation directly alter the input representation, potentially leading to noticeable shifts in downstream classification performance. In contrast, contextual embeddings, like RoBERTa, generate word representations that adapt dynamically based on the surrounding words in a sentence. It allows contextual models to better capture meaning and makes them more resilient to the perturbations caused by back-translation, often yielding semantically consistent representations despite surface-level variation [19,20]. A wealth of research supports this assertion, demonstrating the superior ability of contextual embeddings to capture and detect subtle meaning shifts, a critical factor when analyzing divergence in back-translation. For instance, studies [21,22] have shown that contextualized representations generally outperform static embeddings in identifying meaning shifts and recovering implicit information, as they can adapt to a word’s specific context. This contextual sensitivity is effective at discerning changes arising from lexical composition or nominal coercion, areas where static models may fall short or only detect shifts in limited scenarios [21,23]. The very design of dynamic contextualized embeddings, which excel at capturing word meaning variability across linguistic and extralinguistic contexts, underpins their suitability for tasks involving semantic flexibility, like maintaining fidelity through back-translation [22]. Therefore, we anticipate that the impact of back-translation will be more pronounced for static embeddings, resulting in greater divergence from the original data, and considerably limited for contextual embeddings, owing to the latter’s inherent contextual adaptability and demonstrated capacity for robust semantic representation.

This paper is structured as follows: the *Related Work* section reviews prior studies relevant to the use of back-translation and text data augmentation in natural language processing tasks. The *Materials and Methods* section describes the Slovak text corpora used, the training of both static and contextual embedding models, the preparation of classification datasets, and the back-translation procedure, followed by a detailed description of the classification experiments. The *Results* section presents the performance outcomes of the models trained on original and augmented datasets and analyzes the impact of back-translation on topic classification performance.

### Related work

One of the fields of natural language processing is machine translation. Machine translation, whether statistical machine translation (SMT) or neural machine translation (NMT), requires the availability of high-quality parallel data, which can be a challenge for low-resource languages. Several studies have shown that back-translation can be useful in this case, as discussed in the following paragraphs.

An interesting study [24] was conducted to find out the impact of back-translation from 108 languages to English on embeddings. The embedding space varies consistently across all 108 languages tested. Languages that yield the furthest embeddings, which are considered better for generalization, often correspond to those with poorer translation quality as measured by BLEU scores. Interestingly, the study found that languages with worse translation quality (lower BLEU scores) tend to produce embeddings that are more beneficial for generalization.

The authors [25] used human English – German translations to train the basic NMT system, which they supplemented with synthetic translations using reverse translation. The addition of synthetic data proved effective for training the NMT model. Authors [26,27] dealing with the same language pair came to a similar conclusion. By using back-translation for the purpose of augmenting the training data, they improved the performance of the NMT system. Similarly, authors [28] successfully improved translation accuracy using back-translation from high-resource language, namely Russian to improve machine translation for a low-resource language Belarusian – English.

According to recent study [3] text classification is a fundamental task in NLP. An interesting study [29] investigated the effect of data augmentation on accuracy in text classification of IMDB movie reviews dataset, while the accuracy classification model was the Universal Language Model Fine-tuning (ULMFiT) [30]. This model is based on the Long Short-Term Memory (LSTM) architecture and produces contextual representations of words. The authors compared the impact of two techniques, namely random token perturbation introduced by the authors [3] and back-translation. The study was carried out on low-resource sources and interesting results were achieved. While back-translation brought a significant improvement with a limited amount of data, the random token perturbation technique essentially did not bring a significant improvement. The impact of the back-translation technique on text classification was also dealt with in a study [31] conducted on three Chinese datasets on the sentiment of reviews from different domains. The authors used the Tokenizer function from the Keras library to create the vectors. Individual sequences were subsequently input to LSTM and CNN models. Back-translation technique helped to improve the classification accuracy by 2.3%. Other study [32] focused on enhancing the sentiment classification power for Marathi language by leveraging augmentation techniques including back-translation. The authors used two datasetets (in-domain and cross-domain) and they used BERT based model. The authors achieved performance improvement.

An interesting point of view was obtained in study dealing with back-translation in low-resource text classification scenarios [29]. While this paper primarily showcases significant gains for backtranslation in low-resource text classification scenarios, it also explicitly demonstrates that the benefits of training with backtranslated examples decrease as the size of the available training data increases. For instance, when using the ULMFit model, the F1-score improvement from backtranslation ranged from 4.2% for very small datasets (50 examples) down to 0.4% for larger datasets (10,000 examples). Although ULMFit is a model that leverages pre-trained language models (which typically involve contextual embeddings), the principle of saturation – where additional data provides increasingly smaller gains as performance approaches an asymptote – is generalizable. This concept applies to models using static word embeddings as well, especially when the original dataset is sufficiently large or the baseline model is already strong. While back-translation often demonstrates notable improvements, especially in low-resource settings, some studies [29,31] suggest that its impact on classification performance can be minimal or exhibit diminishing returns under certain conditions. For instance, research indicates that the benefits of back-translation tend to decrease as the size of the original training data increases. When a sufficiently large dataset is already available, the additional, synthetically generated examples may introduce only marginal new information, leading to negligible F1-score improvements over a strong baseline [33]. Furthermore, the quality of the back-translation process itself plays a crucial role. If the machine translation systems used are not highly accurate for the specific language pair or domain, the generated synthetic data might contain noise or semantic inaccuracies, which can limit or even counteract the potential benefits for the downstream classification task [34].

In the Introduction section, we stated that the text needs to be represented before entering the model, and in the case of text representation at the level of words, we distinguish between static word embeddings and contextual word embeddings. The classification model can therefore be trained on sequences and corresponding labels, and in the case of unknown data, the input to the model is the sequence itself. The above-mentioned studies show that data augmentation using the back-translation technique makes sense in terms of improving the performance of classification models, even if in some cases the improvement amounts to only a few percentage points. The aim of our study is to look at the differences in the effect of back-translation augmentation using static vectors and using contextual vectors.

## Materials and methods

**Fig 1:**
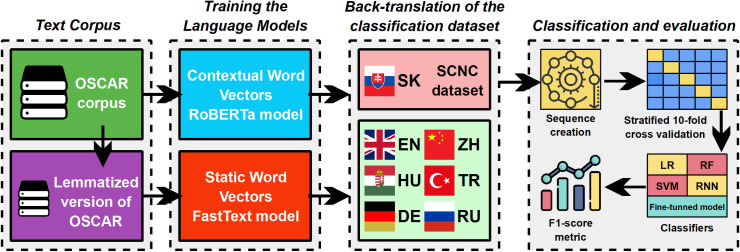
Methodology steps.

This paper focuses on analyzing the impact of a specific text data augmentation technique, namely back-translation, in the task of topic classification in the Slovak language. Our previous work [35] examined the effects of back-translation and other text data augmentation methods on the quality of word vectors generated by the Word2Vec Skip-gram model [12] for English. However, in this study, we do not aim to improve the quality of word vectors themselves. Instead, we investigated the influence of the back-translation technique on expanding classification datasets and its effect on the performance of topic classification. Additionally, we analyze the differences in the effectiveness of this technique between a model that generates static word vectors and a model utilizing contextual word vectors based on the Transformer architecture [36]. The research methodology (Fig 1) is divided into several key steps to ensure a thorough examination of the effects under investigation:

1. **Text corpus for training language models:** Selection and preprocessing of a Slovak-language text corpus.
2. **Training the model for static word vectors:** The FastText model [13], based on the Continuous Bag of Words (CBOW) architecture, was trained on the preprocessed corpus.
3. **Training the model for contextual word vectors:** The RoBERTa model [17], utilizing the Transformer architecture, was trained on the same corpus.
4. **Dataset for topic classification:** Selection and preprocessing of the dataset for topic classification in the Slovak language.
5. **Back-translation of the classification dataset:** The classification dataset was translated into several selected languages, and the resulting translations were then back-translated into Slovak.
6. **Classification and evaluation:** Topic classification was performed for both models (FastText and RoBERTa) on the original and back-translated datasets. The results were evaluated using stratified 10-fold cross-validation. We specifically analyzed the results for the original dataset as well as for each version of the dataset created through back-translation.

### Text corpus for training language models

The first step in our methodology involved identifying and preprocessing a sufficiently large text corpus to train the selected language models. We chose the OSCAR corpus (Open Super-large Crawled Aggregated coRpus), which contains texts for 166 languages, including Slovak. For our study, we used the 2109 version of OSCAR [37], derived from the Common Crawl corpus. Only the Slovak portion of this corpus was used. The preprocessing of the corpus involved several stages:

- **Removal of non-Slovak text segments:** All text segments not in the Slovak language were excluded.
- **Removal of non-Slovak characters:** We eliminated characters such as mathematical symbols, pictograms, emoticons, and repeated special characters and spaces.
- **Removal of duplicate and similar sentences:** Sentences that were repeated or very similar were removed. URL addresses were replaced with the string “<url>”.
- **Removal of text within parentheses:** All content enclosed in parentheses was deleted.
- **Selection of medium-length paragraphs:** Medium-length paragraphs were selected for further processing. Extremely short paragraphs would not provide the model with sufficient context to learn meaningful language representations, while overly long paragraphs would exceed the maximum sequence length of 256 tokens allowed by our contextual model (RoBERTa). Therefore, we selected paragraphs with a length between 300 and 1300 characters, which corresponds approximately to 64–256 tokens, ensuring optimal compatibility with the model architecture and input requirements.
- **Removal of similar paragraphs:** Text paragraphs with at least 50% similarity to previous paragraphs were eliminated, as the initial duplicate removal was not sufficient.
- **Lowercasing:** All text in the corpus was converted to lowercase to ensure consistency across the dataset.

After thoroughly cleaning the corpus, we created a subset intended for training language models. Since our goal was not to develop language models that outperform state-of-the-art models, a smaller corpus was considered sufficient. The resulting subset contained approximately 900,000 text paragraphs, comprising 65 million words, of which more than one million were unique words.

This preprocessed text was suitable for training contextual language models, such as RoBERTa, which rely on preserving the sentence structure and context. However, for the static word embedding model FastText, we applied additional preprocessing. Specifically, we tokenized the text into individual words, removed punctuation, filtered out stop words, and lemmatized the remaining words to ensure that each term was represented in its base form.

### Training the model for static vectors

We acknowledge that freely available static word representations currently exist for Slovak; however, our goal was to examine and compare the impact of the back-translation technique on both static and contextual word embeddings. Therefore, we decided to train both types of embeddings on the same text corpus to ensure that the results of the study would be comparable.

For creating static word representations, we selected the FastText model [13], which is particularly suitable for languages with rich morphology, such as Slovak. FastText is inspired by the Word2Vec model [12] but offers significant improvements by working on the level of subword units, generated through character n-grams. For instance, the word “vector” can be split into trigrams (n = 3): “vec”, “ect”, “cto”, “tor”. Each of these trigrams has its own vector, and the final word representation is the average of these trigrams, which helps to capture the meaning of the word more effectively.

FastText supports two main architectures for learning word embeddings: CBOW (Continuous Bag of Words) and Skip-gram, similar to Word2Vec. In the CBOW architecture, the model attempts to predict the current subword based on its context, i.e., the surrounding subword units. This architecture is particularly effective for learning embeddings for frequently occurring words. On the other hand, the Skip-gram architecture focuses on predicting the contextual subwords based on the current subword. This architecture is more suitable for training representations of less frequent words that appear in specific contexts.

In training our FastText model, we were guided by the original parameters proposed by the FastText authors, with some slight modifications. We chose the CBOW architecture, set the vector dimension to 250, the context window size to 5, the number of n-grams to 2, the learning rate to 0.05, and the number of training epochs to 30.

### Training the model for contextual vectors

To ensure a consistent comparison between static and contextual word representations, we decided to train our own contextual language model, despite the availability of monolingual models for Slovak, such as SlovakBERT [38]. For this purpose, we selected a model based on the Transformer architecture [36], specifically RoBERTa (Robustly Optimized BERT Pretraining Approach) [17], which enhances the original BERT model [15] with several improvements. One key difference is the use of dynamic masking, which enables the model to capture context more effectively and improves performance across various natural language processing tasks.

RoBERTa, like other Transformer-based models, leverages the self-attention mechanism, which allows the model to selectively focus on different parts of the input sequence. This mechanism enhances the model’s ability to understand the meaning of individual subwords (tokens) within their broader context. Transformer architecture consists of two main components: encoder and decoder layers. RoBERTa, however, only utilizes the encoder since its goal is to generate contextualized vector representations of tokens, without producing output sequences as is typical for models with decoders.

Each encoder layer comprises multiple multi-head attention mechanisms, allowing the model to simultaneously track various parts of the text. These heads operate in parallel, improving the model’s ability to capture dependencies between tokens. Subsequently, fully connected layers transform these inputs into vector representations that encode the semantic properties of each token in the context of the entire sequence.

For pretraining our RoBERTa model, we used libraries from HuggingFace [39], with a tokenization method based on Byte-Pair Encoding (BPE) and a vocabulary size of 50,264 tokens. Training was conducted on the same text corpus as our FastText model, ensuring data consistency. Our model contained 6 encoder layers, 12 attention heads, and a hidden size of 576, with a total of 58 million parameters. The maximum sequence length was set to 256 tokens. The model was trained with a batch size of 128 over 30 epochs. For optimization, we used the Adam optimizer with a learning rate of 0.0001, dropout set to 0.1, and weight decay of 0.01.

### Dataset for topic classification

The Slovak language is currently classified as a low-resource language [18], meaning there is a limited availability of datasets, including those intended for classification tasks, as well as for other areas of natural language processing (NLP). In this study, we aim to examine the impact of the back-translation technique, which could significantly contribute to solving various NLP tasks for Slovak. To evaluate the effects of this technique, we selected a news topic classification task, utilizing the *Slovak Categorized News Corpus* [40]. This dataset contains news articles categorized into six themes: Economy, Culture, Politics, World, Sport, and Health. The files in this dataset are accompanied by detailed annotations, including information on tokenization, sentence boundary identification, stop words, morphological analysis, named entity recognition, and lemmatization.

However, the dataset itself did not contain the original continuous texts, which necessitated preprocessing to reconstruct the continuous text from individual annotated files. After this reconstruction, we obtained over 86,000 sentences, with each sentence labeled according to its corresponding thematic category. We identified and removed several duplicates within the dataset. Since each file contained multiple sentences, we combined them into single records with a maximum length of 500 characters. After preprocessing, the dataset comprised a total of 14,547 records, categorized as follows:

- Health (0): 1,076 records
- Politics (1): 5,200 records
- Sport (2): 3,338 records
- World (3): 1,122 records
- Economy (4): 3,704 records
- Culture (5): 107 records

To ensure dataset balance, we selected exactly 1,000 records from each category, excluding the Culture (5) category due to its low representation.

### Back-translation of the classification dataset

Back-translation is one of the most straightforward techniques to implement among text data augmentation methods, as it does not require access to specialized resources like synonym databases [1]. In contrast, synonym replacement does require such resources. For example, in the case of static word embeddings, where all words are lemmatized, replacing a word with its synonym is relatively simple, as it involves merely substituting one lemma for another. However, for contextual word embeddings, this process becomes more complex due to the continuous nature of the generated token sequences. In such cases, the synonym must also match the correct grammatical form, which significantly increases the complexity of synonym replacement.

The effectiveness and complexity of text augmentation techniques can also be influenced by the morphological structure of the target language. Morphology examines how word forms change, their meanings, parts of speech, and grammatical categories. Languages differ in how they encode grammatical information, and these differences can affect how well methods like synonym replacement or back-translation perform. For instance, in fusional or agglutinative languages, where a single word form may carry multiple grammatical meanings or consist of several morphemes, generating grammatically correct and semantically equivalent augmentations becomes more challenging. This is especially true when using static embeddings, which do not capture context. Conversely, analytical languages with simpler word forms may be more amenable to such techniques.

Based on morphological characteristics, languages can be broadly categorized into analytical and synthetic types. Synthetic languages are further divided into agglutinative, fusional, and other subtypes. Therefore, in our study, we focused on three prominent morphological types: analytical, agglutinative, and fusional languages. These are widely distributed across different regions and constitute major linguistic groups [41,42]:

- Analytical languages (modern English and Mandarin Chinese) are characterized by simple, unchangeable word forms, where grammatical relationships are primarily expressed through word order and function words.
- Agglutinative languages (Hungarian and Turkish) use affixes (prefixes and suffixes) that clearly attach to a root word, with each morpheme carrying a specific grammatical meaning. This morphological structure is typical of languages in Europe, Asia, and other regions.
- Fusional languages (German and Russian) have a complex system of declension and conjugation, where a single morpheme can simultaneously express multiple grammatical features. Slovak also belongs to this group and is the language of the original classification dataset [42].

The selection of these three morphological types was motivated by their potential impact on the effectiveness of back-translation as a data augmentation strategy. Since back-translation relies on machine-generated paraphrases of original texts, languages with different morphological structures may pose varying levels of difficulty for generating grammatically correct and semantically faithful translations. For example, in fusional languages, where a single morpheme can carry several grammatical features, errors in translation may propagate more easily, reducing the quality of augmentation. In contrast, analytical languages, with minimal morphological marking, may yield cleaner and more consistent translations. By systematically comparing back-translation performance across these language types, we aim to determine whether morphological typology affects the utility of this augmentation method. This approach also allows us to examine whether the observed differences can be attributed to linguistic structure or are more likely caused by translation quality and model limitations.

For each of these morphological types, we selected two representative languages into which we translated the classification dataset, followed by back-translation into Slovak. This resulted in six back-translated classification datasets. The selection of two languages per morphological type reflects a balance between diversity and feasibility. Using two languages allows for capturing variation within each type, while avoiding the computational and logistical overhead of a larger-scale multilingual setup. The specific languages were chosen to represent both linguistic diversity and geographical distribution. For analytical languages, we selected English, as a widely used global language, and Mandarin Chinese, due to its structural contrast and worldwide prevalence. In the agglutinative group, Hungarian and Turkish were chosen as prominent representatives of this morphological system within Europe and Asia. For fusional languages, we included German and Russian, both of which are typologically rich and widely spoken in different regions. The translation of the original dataset was carried out using the Google Cloud Translation tool. In addition, for the FastText model, it was necessary to lemmatize the texts of both the original and the back-translated datasets.

To further understand potential sources of variation in performance, we also evaluated the quality of machine translation used during the back-translation process. We calculated the arithmetic mean of BLEU and METEOR scores between the original and back-translated texts for each language. The results (Table 1) show considerable differences in translation quality across languages, with English achieving the highest scores (BLEU: 0.5349, METEOR: 0.7826) and Chinese the lowest (BLEU: 0.2585, METEOR: 0.5444). These disparities suggest that the effectiveness of back-translation may not be solely influenced by morphological type, but also by the quality and consistency of the machine-translated data.

**Table 1:** Evaluation of machine translation quality for back-translated datasets using BLEU and METEOR scores.

Morphological type	Language	BLEU score	METEOR score
Analytical	English	0.5349	0.7826
	Chinese	0.2585	0.5444
Agglutinative	Hungarian	0.3727	0.6540
	Turkish	0.3408	0.6318
Fusional	German	0.4038	0.6799
	Russian	0.3852	0.6635

### Experimental setup and evaluation procedure

Using stratified 10-fold cross-validation, we classified and evaluated the performance of each classification model on all datasets containing both static vectors generated by the FastText model and contextual vectors created by the RoBERTa model. Since text data augmentation techniques aim to enrich datasets rather than simply replace existing data, it is logical that both the original and back-translated records are present during classification. The data splitting process was as follows:

In each fold, we divided the original dataset (with 9 parts used for training and 1 part for evaluation). We applied the same division to the back-translated data of the specific language, ensuring that the training records of the back-translated dataset corresponded to those in the original dataset. The training of the classification model utilized the training records from both the original and back-translated datasets, while the evaluation of the model’s performance was conducted solely on the evaluation records of the original dataset to accurately assess the impact of the back-translation technique. This procedure was applied across all back-translated datasets.

Naturally, the effect of classification on back-translated datasets can only be evaluated if we also create a classification model for the original data. For baseline comparison, we used only the original training records and evaluated classification accuracy on the evaluation records using stratified 10-fold cross-validation. We labeled this evaluation as *original_single*. Since this approach trains the classifier solely on the original records, while other models are trained on a combination of original and back-translated data (thus doubling the number of records), we introduced the *original_duplicated* method, where the classifier is trained on duplicated original training data for comparison.

**Fig 2:**
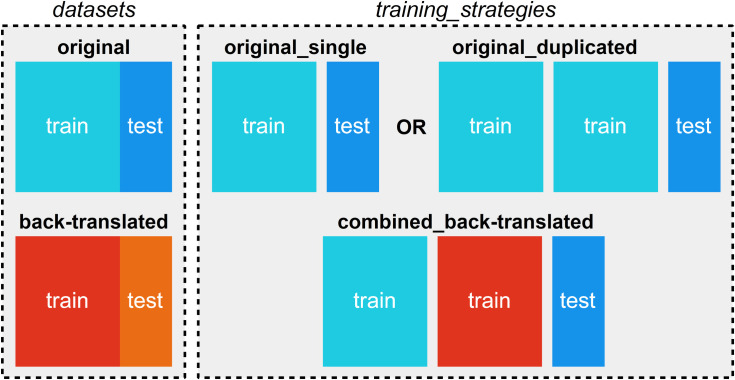
Training strategy.

These different training approaches, referred to collectively as *training_strategies*, include *original_single*, *original_duplicated*, and *combined_back-translated*, the latter of which involves training on both the original and back-translated training datasets. The evaluation for all strategies is performed using the original test set. This training strategy is illustrated in Fig 2 and is consistently applied across each fold.

The complete pseudocode of the unified classification pipeline, which includes data splitting, training strategies, input preparation, and model evaluation, is provided in S1 Algorithm.

### Classification models with static word embeddings

Before performing classification, it was necessary to transform the textual data into classification sequences that align with the requirements of the chosen classification algorithms. In our research, we employed Logistic Regression, Random Forest, Support Vector Machine (SVM), and RNN-LSTM algorithms. These models were selected because they are commonly used in text classification tasks and allow us to compare traditional machine learning methods with a neural approach. We also based this choice on our previous work [35], where similar models were applied in a related setting. Most of these models, except for RNN-LSTM, require input data in a 2D format, whereas RNN-LSTM requires input in a 3D format.

For each dataset, we obtained a vector representation for every lemmatized word in each record. This vector was calculated as the average of all bigrams of the given word, with each vector having a length of 250. Since the number of words varied across records, this resulted in sequences with different numbers of word vectors. To address this issue, we padded the sequences with zero vectors to a maximum length of 50 vectors.

As classifiers such as Logistic Regression, Random Forest, and SVM require input data in a 2D format, we concatenated the word vectors for each record into a single sequence. The resulting sequences had a dimensionality of 250 x 50 = 12,500. The second dimension represented the number of records in the respective dataset.

For the classification tasks, we employed machine learning algorithms available in the Scikit-learn library, each configured with specific parameters. The Logistic Regression classifier was initialized with a maximum of 2000 iterations. The Random Forest model was set to use 200 trees (estimators), while the Support Vector Machine (SVM) classifier was configured with a maximum of 2000 iterations. All other parameters for these algorithms were kept at their default settings.

In addition to traditional machine learning models, we implemented a deep learning approach using an RNN-LSTM model from the TensorFlow Keras library. The architecture started with an input layer shaped to match the training data in 3D, i.e., sequences composed of 50 elements, where each element was a vector of dimension 250. The LSTM model consisted of two layers: the first LSTM layer had 64 units and was set to return sequences, followed by a dropout layer with a rate of 0.1 to prevent overfitting. The second LSTM layer had 32 units, followed by another dropout layer. Finally, a dense layer with a SoftMax activation function was added to classify the data into the appropriate number of topic categories. The classifier was trained for a maximum of 15 epochs, with early stopping based on evaluation loss.

### Classification models with contextual word embeddings

Unlike static vectors, where words in the text were lemmatized and the text contained no punctuation marks, contextual vectors work with the entire continuous text during classification. This continuous text is tokenized, and the tokens from the sequence are fed into the model, which classifies the topic category based on them. The classification of contextual vectors is more challenging because there is no single vector for each word; instead, a vector is generated for each token depending on the context in which it appears. A widely adopted approach for this is fine-tuning a pre-trained model, allowing for the evaluation of the model’s classification performance. A classification layer is added to the model, mapping its output to a specific number of classes. The optimization process adjusts the weights of the entire model, not just the added layer. When fine-tuning the model for a classification task, tokenized sequences are provided as input along with the corresponding topic categories.

The results were evaluated using stratified 10-fold cross-validation with the same training strategy. The learning rate was set to 1 × 10^−5^, and the batch size was 64 sequences. The models were fine-tuned for up to 15 epochs, with early stopping based on evaluation loss.

### Performance evaluation metric

The primary evaluation metric for model performance was the F1-score, which provides a balanced measure of precision and recall. Precision is the ratio of true positive results (TP) to the sum of true positives and false positives (TP + FP), while recall measures the ratio of true positives relative to the total number of true positives and false negatives (TP + FN). The F1-score, as the harmonic mean of precision and recall, takes both false positive and false negative results into account, offering a comprehensive evaluation of the classifier’s performance.


$$F1\;score\;=2\times \frac{Precision\times Recall}{Precision+Recall}$$ (1)

### Computational resources and runtime analysis

All experiments with traditional machine learning models (Logistic Regression, Random Forest, and Support Vector Machine) and the RNN-LSTM model were conducted on a CPU-based server equipped with an 64-core AMD EPYC 7542 processor. The RoBERTa model was fine-tuned on an NVIDIA A100 GPU with 40 GB of memory.

To improve training efficiency, both deep learning models (RNN-LSTM and RoBERTa) were trained with early stopping based on evaluation loss. On average, RoBERTa required 8 epochs and RNN-LSTM 7 epochs to converge. The average training time per fold for each model and strategy is summarized in Table 2.

**Table 2:** Average runtime per fold (in seconds) for each model and training strategy.

Model	Hardware	Strategy	Average time per fold (s)
Logistic Regression	CPU (EPYC 7542)	original_single	61.2
		original_duplicated/ combined_back-translated	242.4
Random Forest	CPU (EPYC 7542)	original_single	3.6
		original_duplicated/ combined_back-translated	7.9
SVM	CPU (EPYC 7542)	original_single	605.8
		original_duplicated/ combined_back-translated	1501.6
RNN-LSTM	CPU (EPYC 7542)	original_single	35.1
		original_duplicated/ combined_back-translated	70.2
RoBERTa	GPU (A100 40GB)	original_single	119.7
		original_duplicated/ combined_back-translated	239.8

## Results

In this section, we present the results of our classification experiments, divided into two parts corresponding to the two types of word representations used in the study. First, we show the performance of models using static word vectors (FastText), followed by results obtained with contextual word embeddings (RoBERTa).

### Evaluation of results for static vectors

This subsection presents the classification results obtained using static word embeddings (FastText). We compare the performance of four classification models under different training strategies, focusing on how back-translated data in various languages affect classification performance.

**Fig 3:**
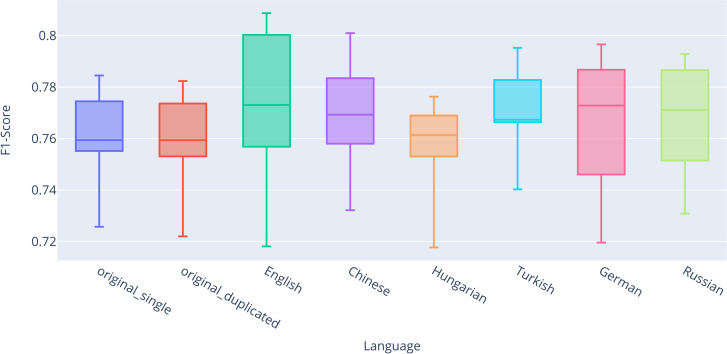
Box plot comparing F1-scores for the Logistic Regression classifier.

**Fig 4:**
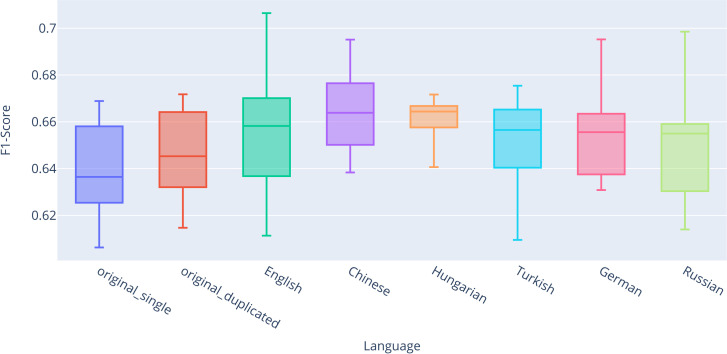
Box plot comparing F1-scores for the Random Forest classifier.

**Fig 5:**
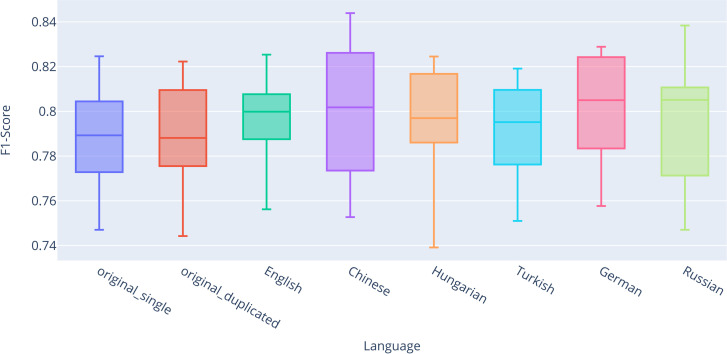
Box plot comparing F1-scores for the Support Vector Machine classifier.

**Fig 6:**
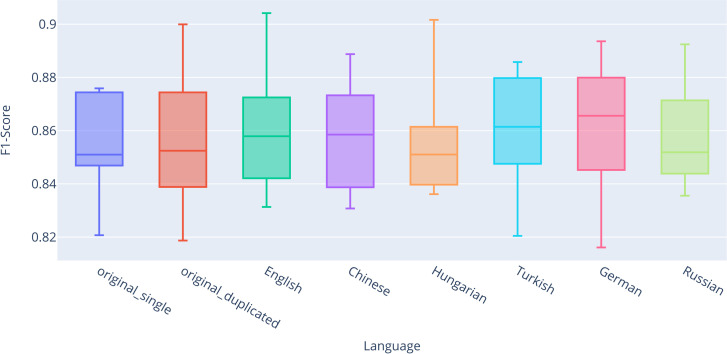
Box plot comparing F1-scores for the RNN-LSTM classifier.

For completeness and transparency, S1 Table presents the exact values of descriptive statistics for all classifiers across all datasets, evaluated using stratified 10-fold cross-validation. For better visual representation, the results are also summarized in box plots (Figs 3–6). Based on the results, significant performance differences between classifiers are evident. The best-performing model was RNN-LSTM, followed by SVC and Logistic Regression, with Random Forest yielding the weakest results.

We can observe the impact of the back-translation technique on classification with Logistic Regression (Fig 3). It is important to note that there is no significant difference between *original_single* (Q2 = 0.7594) and *original_duplicated* (Q2 = 0.7594), indicating that the quantity of training data does not substantially affect classification performance. The most notable differences in the median were observed for the combined back-translated datasets in English (Q2 = 0.7730, + 0.0136 compared to *original_single*), German (Q2 = 0.7728, + 0.0134), and Russian (Q2 = 0.7711, + 0.0117). Overall, for each language, we achieved higher median and mean values compared to both original classifications.

To statistically verify the impact of the back-translation technique on classification, we employed the Wilcoxon matched pairs test. This non-parametric test serves as a suitable alternative to the t-test for dependent samples, without requiring the data to follow a normal distribution, which is especially advantageous for small sample sizes. The test examines whether the differences in F1-scores between two groups (*original_single* and *combined_back-translated* for all languages) are statistically significant.

We establish the null hypothesis (H_0_), which asserts that the back-translation technique has no effect on the F1-score, meaning the differences between the groups are not significant. Should the null hypothesis be rejected, the alternative hypothesis (H_1_) is accepted, which suggests that the back-translation technique has a statistically significant positive impact on classification. A p-value below 0.05 indicates the rejection of the null hypothesis, thereby confirming the effectiveness of the back-translation technique in enhancing classifier performance.

Since multiple pairwise comparisons were conducted (one for each language), we applied a correction for multiple testing to control the false discovery rate (FDR) and reduce the likelihood of Type I errors. Specifically, we used the Benjamini-Hochberg correction [43], which is commonly recommended when testing multiple hypotheses in parallel. The corrected p-values are reported in Table 1 under the column FDR-corrected p-value.

The Wilcoxon paired test revealed statistically significant differences in classification performance using the back-translation technique only for certain languages. Specifically, the test showed a significant positive impact of back-translation on English, Chinese, and Turkish, even after FDR correction. In the case of Hungarian, German, and Russian, no statistically significant differences were observed, despite the fact that German and Russian reached relatively high F1-scores (Table 3).

**Table 3:** Wilcoxon matched pairs test (Logistic Regression classifier)*.*

Pair of Variables	Wilcoxon Matched Pairs Test Marked tests are significant at p < 0.05				
	Valid N	T	Z	*p*-value	FDR-corrected *p*-value
original_single & English	10	4	0.20386	0.01367	0.02734
original_single & Chinese	10	1	0.05096	0.00391	0.01172
original_single & Hungarian	10	20	1.01929	0.49219	0.49219
original_single & Turkish	10	1	0.05096	0.00391	0.01172
original_single & German	10	11	0.56061	0.10547	0.12656
original_single & Russian	10	9	0.45868	0.06445	0.09668

We can also observe the impact of the back-translation technique on classification using Random Forest (Fig 4), which performed the worst among all classifiers. Unlike the results for logistic regression, the difference between *original_single* (Q2 = 0.6364) and *original_duplicated* (Q2 = 0.6453) is evident for Random Forest. The most pronounced differences in median values were observed for the *combined_back-translated* datasets in Hungarian (Q2 = 0.6644, + 0.0280 compared to *original_single*), Chinese (Q2 = 0.6639, + 0.0275), and English (Q2 = 0.6582, + 0.0218). Overall, for each language, we again achieved higher median and mean values compared to both original classifications.

For the Random Forest classifier, the results of the Wilcoxon paired test indicate statistically significant differences in the impact of the back-translation technique for certain languages (Table 4). Notably, significant improvements were observed for Chinese, and Hungarian, with both remaining significant after FDR correction. As in the previous analysis, p-values were adjusted for multiple testing using the Benjamini-Hochberg correction. Although the uncorrected p-value for German was below 0.05, the effect was no longer significant after correction. For English, Turkish, and Russian, no statistically significant differences were found.

**Table 4:** Wilcoxon matched pairs test (Random Forest classifier)*.*

Pair of Variables	Wilcoxon Matched Pairs Test Marked tests are significant at p < 0.05				
	Valid N	T	Z	*p*-value	FDR-corrected *p*-value
original_single & English	10	9	0.45868	0.06445	0.09668
original_single & Chinese	10	4	0.20386	0.01367	0.04102
original_single & Hungarian	10	3	0.15289	0.00977	0.04102
original_single & Turkish	10	16	0.81544	0.27539	0.27539
original_single & German	10	7	0.35675	0.03711	0.07422
original_single & Russian	10	10	0.50965	0.08398	0.10078

The second-best classification results were achieved using the SVM classifier (Fig 5). Similar to the Logistic Regression classifier, the differences between the *original_single* (Q2 = 0.7893) and *original_duplicated* (Q2 = 0.7881) datasets were not significant, with slightly worse outcomes observed for the duplicated original dataset. The most notable differences in median values were found in the *combined_back-translated* datasets for Russian (Q2 = 0.8051, + 0.0158 compared to *original_single*), German (Q2 = 0.8050, + 0.0157), and Chinese (Q2 = 0.8018, + 0.0125). Overall, for each language, we consistently achieved higher median and mean values compared to both original classification datasets.

The Wilcoxon paired test for the SVM classifier demonstrates that the strong classification results are also statistically supported for most languages (Table 5). Before applying FDR correction, significant improvements were observed for English, Chinese, Turkish, German, and Russian. However, after applying the Benjamini-Hochberg correction for multiple comparisons, only English and German remained statistically significant at the 0.05 threshold. Although some comparisons (e.g., Chinese, Turkish, Russian) no longer meet the strict significance threshold after correction, it is worth noting that their uncorrected and corrected p-values remain relatively low (typically between 0.05 and 0.06). This suggests that the positive impact of the back-translation technique in these languages is likely meaningful, even if not formally significant after controlling for the false discovery rate. The only language for which the back-translation technique showed no statistical support was Hungarian, where both uncorrected and corrected p-values exceeded the significance threshold.

**Table 5:** Wilcoxon matched pairs test (Support Vector Machine classifier)*.*

Pair of Variables	Wilcoxon Matched Pairs Test Marked tests are significant at p < 0.05				
	Valid N	T	Z	*p*-value	FDR-corrected *p*-value
original_single & English	10	1	0.05096	0.00391	0.02344
original_single & Chinese	10	7	0.35675	0.03711	0.05566
original_single & Hungarian	10	9	0.45868	0.06445	0.06445
original_single & Turkish	10	7	0.35675	0.03711	0.05566
original_single & German	10	3	0.15289	0.00977	0.02929
original_single & Russian	10	8	0.40772	0.04883	0.05858

The final classification model analyzed in relation to the impact of the back-translation technique for static vectors is the RNN-LSTM, as illustrated by the box plot in Fig 6. While this model demonstrated the highest classification performance overall, its effectiveness in leveraging the back-translation technique was among the lowest. The difference between *original_single* (Q2 = 0.8510) and *original_duplicated* (Q2 = 0.8525) is minor but notable when compared to other languages. Although the results for all languages outperformed *original_single* in terms of median values, they did not surpass *original_duplicated* for Hungarian (Q2 = 0.8510) and Russian (Q2 = 0.8519). The most substantial improvements were observed in German (Q2 = 0.8656, + 0.0146) and Turkish (Q2 = 0.8615, + 0.0105).

The impact of the back-translation technique on the RNN-LSTM classifier was minimal, as confirmed by the results of the Wilcoxon matched pairs test (Table 6). No statistically significant differences were observed for any language, regardless of whether raw or FDR-corrected p-values were considered, indicating that the back-translation technique had a negligible effect on classification results for this model.

**Table 6:** Wilcoxon matched pairs test (RNN-LSTM classifier)*.*

Pair of Variables	Wilcoxon Matched Pairs Test Marked tests are significant at p < 0.05				
	Valid N	T	Z	*p*-value	FDR-corrected *p*-value
original_single & English	10	18	0.91736	0.37500	0.73828
original_single & Chinese	10	20	1.01929	0.49219	0.73828
original_single & Hungarian	10	25	1.27412	0.84570	0.84570
original_single & Turkish	10	12	0.61158	0.13086	0.39258
original_single & German	10	12	0.61158	0.13086	0.39258
original_single & Russian	10	24	1.22315	0.76953	0.84570

### Evaluation of results for contextual vectors

This subsection presents the classification results obtained using contextual word embeddings (RoBERTa). We evaluate the impact of various training strategies on model performance across different datasets, following the same experimental setup as in the previous section. For completeness, we present the results of the descriptive statistics (Table 7) for the classification after fine-tuning the RoBERTa model across all datasets, evaluated using stratified 10-fold cross-validation. Based on these results, particularly with regard to the mean and median values of the F1-score metric, there are no significant performance differences observed between the individual classifications.

**Table 7:** Descriptive statistics of classifier with the RoBERTa model performance on original and back-translation datasets by F1-score metric.

RoBERTa classifier	Count	Mean	Std	Min	25%	50%	75%	Max
**original_single**	10	0.91137	0.01787	0.88243	0.89671	0.91384	0.92546	0.93614
**original_duplicated**	10	0.91303	0.01640	0.88357	0.90107	0.91489	0.92415	0.93593
**English**	10	0.91245	0.01885	0.87969	0.89967	**0.91672**	0.92337	0.94192
**Chinese**	10	0.91232	0.01409	0.89403	0.90025	0.91248	0.92267	0.93592
**Hungarian**	10	0.91287	0.01636	0.88792	0.90169	0.91495	0.92668	0.93415
**Turkish**	10	0.91520	0.01639	0.89192	0.90507	**0.91430**	0.92573	0.94024
**German**	10	0.91360	0.01597	0.89146	0.90270	**0.91584**	0.92373	0.93602
**Russian**	10	0.91147	0.01624	0.88205	0.90213	0.91364	0.91619	0.93798

**Fig 7:**
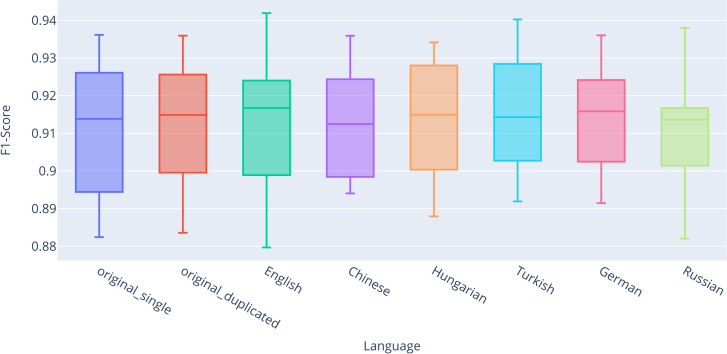
Box plot comparing F1-scores for the fully connected classifier with the RoBERTa model.

The visualization of the results is presented in the box plot (Fig 7), which shows that there is no significant difference between *original_single* (Q2 = 0.9138) and *original_duplicated* (Q2 = 0.9149). What is more interesting, however, is that the improvement in performance for the *combined_back-translated* datasets was observed only for certain languages. The best results were achieved in English (Q2 = 0.9167), German (Q2 = 0.9158), and Turkish (Q2 = 0.9143), while for the remaining languages, the results were either nearly identical or worse compared to *original_single*. It is worth noting that the RoBERTa model achieved the best results among all classifiers compared to static vectors, indicating its superior ability to understand the texts and accurately classify them into categories.

The results of the descriptive statistics confirmed the findings of the Wilcoxon paired test (Table 8). For completeness, p-values were again corrected for multiple comparisons using the Benjamini-Hochberg correction. However, no statistically significant differences were found for any language, neither before nor after correction. This indicates that the back-translation technique had virtually no positive impact on the classification outcomes for this model.

**Table 8:** Wilcoxon matched pairs test (RoBERTa model classifier).

Pair of Variables	Wilcoxon Matched Pairs Test Marked tests are significant at p < 0.05				
	Valid N	T	Z	*p*-value	FDR-corrected *p*-value
original_single & English	10	22	1.12122	0.62500	0.75000
original_single & Chinese	10	21	1.07026	0.55664	0.75000
original_single & Hungarian	10	21	1.07026	0.55664	0.75000
original_single & Turkish	10	16	0.81544	0.27539	0.75000
original_single & German	10	17	0.86640	0.32227	0.75000
original_single & Russian	10	27	1.37605	1.00000	1.00000

## Discussion

Our research experiment aimed to compare the performance of several classifiers trained on datasets augmented with back-translation to those trained on the original datasets, using both static word vectors and contextual word vectors. Our findings clearly demonstrate that back-translation does have a discernible and statistically significant impact on the performance of classifiers utilizing static word embeddings, namely FastText with Logistic Regression, SVM, Random Forest, and RNN-LSTM. Specifically, we observed F1-score improvements ranging up to 2.80% (for Random Forest) across various classifier-language combinations.

These results align directly with our hypothesis, confirming that augmenting data through back-translation can indeed enhance the robustness and performance of models relying on static vector representations. While the observed F1-score improvements for static embeddings were consistently modest (< 2.80%), their statistical significance across multiple classifiers and languages indicates a reliable positive effect. Such modest gains are consistent with observations in other data augmentation studies, particularly when moving beyond extremely low-resource settings. For instance, several studies [29,33] demonstrated that the F1-score improvement from back-translation can diminish significantly, sometimes to less than 1%, as the size of the original training data increases. Our findings for static embeddings, while not reaching the dramatic increases seen in highly data-scarce scenarios, confirm that even these smaller, statistically significant enhancements contribute meaningfully to model performance where static representations are employed, potentially reducing reliance on larger original datasets. Even small, consistent improvements can be valuable in practical applications where every incremental gain contributes to overall system efficacy or competitive advantage according to [29,31]. This contrasts with the effect on contextual embeddings from the RoBERTa model, where back-translation showed negligible impact, with no statistically significant F1-score improvement observed across any language. This highlights a key distinction in how different embedding types respond to this form of data augmentation: models employing powerful, inherently context-aware embeddings are less dependent on such augmentation for performance gains, while static embeddings can still benefit from the expanded data diversity introduced by back-translation, confirming the specific purpose of our investigation.

In general, the results indicate that back-translation tends to improve the classification performance for static word embeddings across most languages. Logistic Regression and Support Vector Machine classifiers showed noticeable improvements when trained on combined datasets (original + back-translated) compared to the original datasets alone. This supports the hypothesis that static embeddings, which are more sensitive to slight lexical changes, benefit from the variation introduced by back-translation.

For the Logistic Regression classifier, the median F1-score increased from 0.7594 (original_single and original_duplicated) to 0.7730 in English, 0.7728 in German, and 0.7711 in Russian when using the combined back-translated datasets. The Wilcoxon test confirmed that these improvements were statistically significant only for English, Chinese, and Turkish, even after applying the FDR correction. This highlights the selective but meaningful impact of back-translation for certain languages.

The SVM classifier achieved the second-best overall performance. The combined back-translated datasets yielded the highest median values in Russian (0.8051), German (0.8050), and Chinese (0.8018). The Wilcoxon paired test indicated statistically significant improvements for English and German after FDR correction. While improvements for Chinese, Turkish, and Russian did not meet the strict significance threshold after correction, their low p-values suggest a likely meaningful effect of back-translation.

Despite its higher overall performance, the RNN-LSTM model exhibited the least responsiveness to back-translation, with negligible improvements. The median F1-score difference between original_single (0.8510) and original_duplicated (0.8525) was minor, and although back-translated datasets outperformed original_single for all languages, they did not surpass original_duplicated for Hungarian (0.8510) and Russian (0.8519). The most substantial improvements were observed in German (0.8656) and Turkish (0.8615). This may suggest that deep learning models like RNN-LSTM, which inherently model sequential data and context, are less dependent on the variations provided by back-translation for static embeddings. This observation aligns with the understanding that recurrent neural networks, by their nature, are designed to model sequential dependencies and contextual information within the data, potentially reducing their reliance on external data augmentation techniques for further performance gains when a robust baseline is already achieved.

The Random Forest classifier performed the worst overall, though it still benefited from back-translation. The most notable improvements in median F1-scores were seen in Hungarian (Q2 = 0.6644), Chinese (Q2 = 0.6639), and English (Q2 = 0.6582). Statistically significant effects (after FDR correction) were confirmed only for Chinese and Hungarian, with other languages showing either marginal or no significance.

Contrary to static embeddings, the impact of back-translation on contextual embeddings was much less significant. The RoBERTa-based model achieved consistently high performance across all datasets, and the addition of back-translated data did not lead to statistically significant improvements. This aligns with the hypothesis that contextual embeddings, which adjust dynamically based on the surrounding text, are more robust to lexical and syntactic variations introduced by back-translation. This is consistent with the design of contextual models, which generate dynamic word representations that are highly sensitive to the surrounding linguistic context [36], rendering them inherently more robust to the variations introduced by data augmentation through back-translation. The RoBERTa model performed exceptionally well, with median F1-scores consistently above 0.91 for all datasets. However, the differences between the original and augmented datasets were minimal. This suggests that RoBERTa’s ability to capture rich contextual information diminishes the need for back-translation as a data augmentation method. While our findings suggest a limited impact of back-translation on RoBERTa’s performance, it is worth noting that some studies have reported performance improvements using back-translation with BERT-based models for certain tasks [32].

Interestingly, the improvements brought by back-translation were not uniform across all languages. In the case of static embeddings, languages like English, Chinese, and Turkish showed significant improvements, while Hungarian and Russian yielded mixed results, showing high F1-scores in some classifiers but lacking consistent statistical support. For contextual embeddings, the performance was more consistent across languages but did not show notable gains from back-translation. These differences may reflect underlying linguistic properties such as morphological complexity or sentence structure. Such variations in effectiveness across languages align with established principles of linguistic typology, which recognize that structural differences, such as morphological complexity and sentence structure [41], can significantly influence the performance of natural language processing techniques. Although fusional languages such as German and Russian exhibit relatively high morphological complexity, only German showed consistent improvements, while Russian did not. This may appear counterintuitive, as one might expect that the morphological richness of fusional languages could provide greater potential for generating diverse and informative paraphrases through back-translation. However, the results suggest that high morphological complexity can also increase the difficulty of producing grammatically correct and semantically accurate augmentations. In the case of Russian, the relatively low translation quality, as indicated by BLEU and METEOR scores, likely reduced the effectiveness of the augmented data. These findings suggest that the impact of back-translation depends on a combination of linguistic features and machine translation quality.

Among the different classifiers tested, RNN-LSTM demonstrated the highest overall performance, although the effect of back-translation was minimal for this model. Logistic Regression and SVM performed well with back-translated data in static embeddings, while Random Forest consistently showed the weakest performance across all experiments. These findings suggest that simpler models, particularly those that do not inherently model the sequential structure of language, may benefit more from data augmentation techniques like back-translation than more complex models like RNNs, which already capture contextual and sequential information from the data itself.

## Conclusion

This study confirms that the back-translation technique can significantly enhance classification performance, especially when using static word vector representations such as FastText, particularly in low-resource languages. In our experiments, classification models like Logistic Regression, Random Forest, and SVM demonstrated significant improvements when trained on combined original and back-translated data. For instance, Logistic Regression classification improved its F1-score from a median of 0.7594 for the original data to 0.7730 when using back-translated data in English and from 0.7594 to 0.7728 in German. In the case of the SVM model, the best results were achieved with a combination of original and back-translated data in Russian and German, where the median F1-score increased from 0.7893 (original) to 0.8051 in Russian and to 0.8050 in German. Conversely, the effect of back-translation was less pronounced and sometimes inconsistent when applied to models utilizing contextual embeddings, such as RoBERTa, which overall achieved the highest performance. While the model’s median F1‑score was consistently above 0.91, improvements from augmentation were marginal, typically in the range of +0.0020 to +0.0030. In some cases, performance remained static or even slightly declined with back-translated data.

Despite these valuable findings, our study has several limitations that warrant consideration. Firstly, our evaluation primarily relied on the F1-score as the sole performance metric. Future work could incorporate additional metrics such as precision, recall, or accuracy to provide a more comprehensive understanding of model performance. Secondly, the scope of this research was confined to topic classification, and the generalizability of these findings to other NLP tasks, such as sentiment analysis or named entity recognition, remains to be explored. Furthermore, while six languages were included, a deeper linguistic analysis of how specific morphological or syntactic features might influence back-translation effectiveness was beyond the scope of this work.

These insights contribute significantly to the understanding of data augmentation strategies in natural language processing, particularly emphasizing the differential impact of back-translation across various embedding types and model complexities.

For researchers working with low-resource languages, back-translation is a valuable augmentation technique, particularly when using static embeddings. However, when working with contextual models, alternative augmentation or adaptation strategies tailored to the specific task may be more effective.

Building upon these findings and addressing the identified limitations, future research could explore several promising directions. Expanding the evaluation to include a wider array of NLP tasks and diverse text domains would enhance the generalizability of back-translation’s efficacy. Investigating the impact of back-translation on a broader spectrum of languages, particularly focusing on specific linguistic characteristics such as varying degrees of morphological richness or syntactic structures, could yield more granular insights. Finally, researching methods to assess and improve the quality of back-translated data for specific language pairs, beyond standard metrics like BLEU or METEOR, could further optimize its application.

## Supporting information

S1 Algorithm Pseudocode of cross-validated classification using original and back-translated datasets. (DOCX)

S1 Table Descriptive statistics of classifier performance on original and back-translated datasets by F1-score metric. (DOCX)
