# Supplementary material for: Back-translation effects on static and contextual word embeddings for topic classification embedding in classification tasks
Source: PLoS One. 2025 Aug 29;20(8):e0330622. doi: 10.1371/journal.pone.0330622 (PMC12396668; doi:10.1371/journal.pone.0330622)
Supplement: S1 Algorithm — (DOCX) [file pone.0330622.s001.docx]

**S1 Algorithm. Pseudocode of cross-validated classification using original and back-translated datasets.**

Algorithm: Cross-validated classification using original and back-translated data

Require:

D_orig ← original dataset (vector or tokenized text sequences)

D_aug_set ← set of back-translated datasets (ENG, GE, HU, CHINA, RU, TU)

K ← number of folds (e.g., K = 10)

Model_type ← one of {static_model, contextual_model}

Strategy ← one of {original_single, original_duplicated, combined_back-translated}

1: for each fold i in stratified K-fold do

2: Split D_orig into D_train_orig[i], D_eval[i]

3:

4: if Strategy == combined_back-translated then

5: for each D_aug in D_aug_set do

6: Align D_train_aug[i] with D_train_orig[i]

7: D_train ← concatenate(D_train_orig[i], D_train_aug[i])

8: else if Strategy == original_duplicated then

9: D_train ← duplicate(D_train_orig[i])

10: else

11: D_train ← D_train_orig[i]

12:

13: if Model_type == static_model then

14: Convert each record into sequence of word vectors

15: Pad each sequence to fixed length (e.g., 50 vectors × 250 dimensions)

16: if model requires 2D input then

17: Flatten each sequence to shape (1 × 12,500)

18:

19: else if Model_type == contextual_model then

20: Tokenize continuous text and generate attention masks

21: Prepare input_ids and attention_mask tensors

22:

23: Initialize model (e.g., LR, SVM, LSTM, RoBERTa) with predefined parameters

24: Train model on D_train (with early stopping if applicable)

25:

26: Predict labels on D_eval

27: Compute evaluation metrics: Accuracy, Precision, Recall, F1-score

28: Store metrics and confusion matrix for current fold

29:

30: Aggregate metrics across all folds

31: Report final performance for each model and training strategy
