# Supplementary material for: Back-translation effects on static and contextual word embeddings for topic classification embedding in classification tasks
Source: PLoS One. 2025 Aug 29;20(8):e0330622. doi: 10.1371/journal.pone.0330622 (PMC12396668; doi:10.1371/journal.pone.0330622)
Supplement: S1 Table — (DOCX) [file pone.0330622.s002.docx]

**S1 Table.** Descriptive statistics of classifier performance on original and back-translated datasets by F1-score metric

| **Logistic Regression** | **Count** | **Mean** | **Std** | **Min** | **25%** | **50%** | **75%** | **Max** |
| --- | --- | --- | --- | --- | --- | --- | --- | --- |
| **original_single** | 10 | 0.75949 | 0.01792 | 0.72571 | 0.75517 | 0.75940 | 0.77298 | 0.78450 |
| **original_duplicated** | 10 | 0.75888 | 0.01832 | 0.72199 | 0.75413 | 0.75936 | 0.77283 | 0.78236 |
| **English** | 10 | 0.77434 | 0.02840 | 0.71811 | 0.75767 | 0.77304 | 0.79800 | 0.80872 |
| **Chinese** | 10 | 0.77089 | 0.02048 | 0.73216 | 0.75864 | 0.76925 | 0.78340 | 0.80094 |
| **Hungarian** | 10 | 0.75746 | 0.01695 | 0.71769 | 0.75314 | 0.76137 | 0.76835 | 0.77626 |
| **Turkish** | 10 | 0.77028 | 0.01662 | 0.74026 | 0.76639 | 0.76730 | 0.78202 | 0.79523 |
| **German** | 10 | 0.76665 | 0.02440 | 0.71956 | 0.74880 | 0.77284 | 0.78564 | 0.79659 |
| **Russian** | 10 | 0.76745 | 0.02306 | 0.73085 | 0.75289 | 0.77111 | 0.78649 | 0.79286 |
| **Random Forest** | **Count** | **Mean** | **Std** | **Min** | **25%** | **50%** | **75%** | **Max** |
| **original_single** | 10 | 0.63900 | 0.02147 | 0.60628 | 0.62631 | 0.63644 | 0.65480 | 0.66887 |
| **original_duplicated** | 10 | 0.64590 | 0.01771 | 0.61470 | 0.63377 | 0.64528 | 0.66080 | 0.67175 |
| **English** | 10 | 0.65525 | 0.02757 | 0.61132 | 0.64052 | 0.65822 | 0.66814 | 0.70646 |
| **Chinese** | 10 | 0.66487 | 0.01707 | 0.63834 | 0.65246 | 0.66385 | 0.67640 | 0.69513 |
| **Hungarian** | 10 | 0.66068 | 0.00968 | 0.64063 | 0.65797 | 0.66438 | 0.66663 | 0.67166 |
| **Turkish** | 10 | 0.65013 | 0.02120 | 0.60951 | 0.64332 | 0.65650 | 0.66403 | 0.67542 |
| **German** | 10 | 0.65479 | 0.02011 | 0.63082 | 0.63809 | 0.65558 | 0.66245 | 0.69522 |
| **Russian** | 10 | 0.65073 | 0.02498 | 0.61397 | 0.63208 | 0.65496 | 0.65890 | 0.69852 |
| **Support Vector Machine** | **Count** | **Mean** | **Std** | **Min** | **25%** | **50%** | **75%** | **Max** |
| **original_single** | 10 | 0.78894 | 0.02224 | 0.74705 | 0.77500 | 0.78928 | 0.80260 | 0.82462 |
| **original_duplicated** | 10 | 0.78790 | 0.02534 | 0.74426 | 0.77557 | 0.78812 | 0.80725 | 0.82228 |
| **English** | 10 | 0.79812 | 0.02014 | 0.75621 | 0.78822 | 0.79986 | 0.80739 | 0.82536 |
| **Chinese** | 10 | 0.79840 | 0.02925 | 0.75273 | 0.77538 | 0.80178 | 0.82075 | 0.84392 |
| **Hungarian** | 10 | 0.79482 | 0.02477 | 0.73915 | 0.78651 | 0.79697 | 0.81358 | 0.82452 |
| **Turkish** | 10 | 0.79351 | 0.02210 | 0.75101 | 0.77975 | 0.79518 | 0.80930 | 0.81912 |
| **German** | 10 | 0.80171 | 0.02281 | 0.75769 | 0.78777 | 0.80500 | 0.82062 | 0.82888 |
| **Russian** | 10 | 0.79737 | 0.03017 | 0.74706 | 0.77594 | 0.80514 | 0.80970 | 0.83838 |
| **RNN-LSTM** | **Count** | **Mean** | **Std** | **Min** | **25%** | **50%** | **75%** | **Max** |
| **original_single** | 10 | 0.85366 | 0.01758 | 0.82071 | 0.84759 | 0.85099 | 0.86902 | 0.87589 |
| **original_duplicated** | 10 | 0.85507 | 0.02441 | 0.81870 | 0.84079 | 0.85245 | 0.87226 | 0.89994 |
| **English** | 10 | 0.86066 | 0.02355 | 0.83133 | 0.84349 | 0.85790 | 0.87245 | 0.90416 |
| **Chinese** | 10 | 0.85836 | 0.02165 | 0.83075 | 0.83916 | 0.85851 | 0.87290 | 0.88875 |
| **Hungarian** | 10 | 0.85562 | 0.01979 | 0.83613 | 0.84145 | 0.85103 | 0.86074 | 0.90163 |
| **Turkish** | 10 | 0.86005 | 0.02105 | 0.82045 | 0.84919 | 0.86145 | 0.87720 | 0.88578 |
| **German** | 10 | 0.86151 | 0.02386 | 0.81611 | 0.84725 | 0.86559 | 0.87921 | 0.89358 |
| **Russian** | 10 | 0.85604 | 0.01769 | 0.83556 | 0.84425 | 0.85188 | 0.86721 | 0.89243 |
